# MicroRNA expression as a prognostic biomarker of tongue squamous cell carcinoma (TSCC): a systematic review and meta-analysis

**DOI:** 10.1186/s12903-024-04182-0

**Published:** 2024-03-31

**Authors:** Yiwei Sun, Yuxiao Li, Wenjuan Zhou, Zhonghao Liu

**Affiliations:** 1 School of Stomatology, Binzhou Medical University, No. 346 The Guanhai Road Yantai, Yantai, Shandong Province 264003 China; 2 The Second School of Clinical Medicine, Binzhou Medical University, No. 346 The Guanhai Road Yantai, Yantai, Shandong Province 264003 China; 3 The affiliated Yantai Stomatological Hospital, Binzhou Medical University, Yantai, 264000 China; 4 Yantai Engineering Research Center for Digital Technology of Stomatology, Yantai, 264000 China; 5 Characteristic Laboratories of Colleges and Universities in Shandong Province for Digital Stomatology, Yantai, 264003 China

**Keywords:** Tongue squamous cell carcinoma, miRNA, Prognostic, Biomarkers

## Abstract

**Background:**

Recent studies have indicated that microRNA (miRNA) expression in tumour tissues has prognostic significance in Tongue squamous cell carcinoma (TSCC) patients. This study explored the possible prognostic value of miRNAs for TSCC based on published research.

**Methods:**

A comprehensive literature search of multiple databases was conducted according to predefined eligibility criteria. Data were extracted from the included studies by two researchers, and HR results were determined based on Kaplan‒Meier curves according to the Tierney method. The Newcastle‒Ottawa Scale (NOS) and GRADE (Grading of Recommendations Assessment, Development, and Evaluation) pro-GDT were applied to assess the quality of all studies. Publication bias was estimated by funnel plot, Egger’s rank correlation test and sensitivity analysis.

**Results:**

Eleven studies (891patients) were included, of which 6 reported up-regulated miRNAs and 7 mentioned down-regulated miRNAs. The pooled hazard ratio (HR) from the prognostic indicator overall survival (OS) was 1.34 (1.25–1.44), *p* < 0.00001, indicating a significant difference in miRNA expression between TSCC patients with better or worse prognosis.

**Conclusion:**

MiRNAs may have high prognostic value and could be used as prognostic biomarkers of TSCC.

## Introduction

Oral squamous cell carcinoma (OSCC) accounts for approximately 90% of all oral malignancies, resulting in the highest morbidity rates in the head and neck region worldwide [1–3]. When compared with OSCC at other sites, tongue squamous cell carcinoma (TSCC), a principal subtype of oral cancer, represents 17.8% of occurrences and shares similarities in etiopathogenesis related to early regional lymph node metastasis [4–7] and malignant proliferation [8, 9]. Notably, despite the application of advanced therapies, the 5-year survival rate and quality of life for TSCC patients are not promising [1, 9]. Compared with the overt symptoms of advanced stage TSCC, assessments such as tissue biopsy are advantageous for identifying patients with early-stage disease and, as a result, increase the chances for better treatment and recovery [10–12]. Thus, in accordance with the World Health Organization (WHO) recommendations for the primacy of early diagnosis and prevention, measures should be taken to achieve early discovery and diagnosis of TSCC with the purpose of prolonging and improving the quality of patients’ lives [10, 13].

According to current clinical practice, the present detection methods for TSCC include but not limited to brush biopsy, CT and MRI scanning, and tissue autofluorescence. Even though some of these applications are widely performed in routine clinical practice, there exist certain limitations, such as labour intensiveness, invasiveness and low sensitivity, that should not be ignored. Recent researches indicate that part of the genetic alterations for TSCC contain progressive change in DNA methylation, overexpression of carcinoembryonic antigen, histone modification and expression level alteration of miRNAs, etc. As a result, the epigenetic alterations, including DNA fragments in saliva [14, 15], immune-related gene transcript [16], the neutrophil-to‐lymphocyte ratio [17], platelet-to-lymphocyte ratio [18] and miRNA expression [19], could be used as the promising biomarkers for early-stage detection for prognosis, which bridge the defect of traditional Tumor-Node-Metastasis (TNM) staging system [20]. In recent years, with the purpose of finding more reliable diagnostic techniques, research based on microRNA molecular mechanisms has become an important academic focus [10]. Studies have reported connections between miRNAs and predicting the prognosis of TSCC, as changes in miRNA expression can reflect the stage of a tumour to some extent.

MicroRNAs (miRNAs) are sophisticated epigenetic molecular markers linked to TSCC patient prognosis. Intrinsically, miRNAs are endogenous, noncoding, single-stranded RNA molecules that are approximately 20–24 nucleotides long and renowned for their conserved gene sequence and distinct expression patterns [21–24]. Generally, miRNAs are characterized by high gene sequence conservation, temporal expression specificity and tissue expression specificity [25]. By mediating dysregulation and translational repression, miRNAs regulate 30% of gene expression in the posttranscriptional stage [24, 25]. During the biological process of TSCC deterioration, miRNAs partly act as oncogenes or suppressor genes in carcinogenesis by dysregulation, as well as having an indirect impact on the expression of proto-oncogenes and cancer suppressor gene [26]. Followed by that, miRNA deteriorate tumour cell proliferation by escaping normal suppressing signals, expediting malignant cell migration and stimulating angiogenesis in tumours [21, 22, 24, 25].

Experiments based on miRNA as a biomarker of malignancy prognosis have made great progress in recent years. However, due to the comparatively high cost and complexity of the process, this technique has not been put into clinical use on a large scale. Distinct from existing meta-analyses of TSCC, this manuscript offers a fresh perspective, bridging the gap between the academic value of miRNAs and their usefulness in clinical applications. This meta-analysis aimed to consolidate and elucidate the prognostic significance of miRNAs in TSCC, paving the way for innovative clinical interventions to enhance patient longevity and quality of life.

## Materials and methods

### Protocol and registration

Following the Preferred Reporting Items for Systematic Review and Meta-analyses (PRISMA) guidelines [27], researchers executed a systematic review encompassing aspects such as protocol, inclusion criteria, search strategy, and outcomes. This systematic review has been catalogued on PROSPERO under the registration number CRD 42,023,391,953.

### Eligibility criteria

Researchers focused on all retrospective cohort studies evaluating the association between variations in miRNA expression and prognostic survival metrics of TSCC. The criteria were based on the PICO elements: participants (patients diagnosed with TSCC), intervention (deregulated miRNA levels in TSCC patients), control (TSCC patients with normal miRNA expression levels), and outcome (prognosis differences in TSCC patients based on miRNA expression variance). Consequently, the PICO question was as follows: is there a difference in prognostic survival indexes between TSCC patients with dysregulation of miRNA expression and those with normal miRNA expression?

The inclusion criteria were studies that reported interrelated prognostic survival indexes, including hazard ratios (HRs), Kaplan–Meier curves and univariate or multivariate Cox regression between TSCC patients with low and high miRNA expression levels.

Regarding the exclusion criteria, non-English studies and those without prognostic survival indexes from which HRs could be extracted or inferred were excluded. Case reports, meta-analyses, systematic reviews, historical reviews and studies at high risk of bias were excluded. Studies that did not elaborate clinical outcomes or that reported content that was not relevant to clinical patients were likewise excluded.

### Sources of information, research and selection

For reference inclusion, relevant articles were identified through literature retrieval from online databases. Specifically, non-English articles were excluded. Databases, including PubMed, Web of Science, Scopus, EMBASE, Cochrane Library and Google Scholar, were searched to retrieve relevant articles focusing on miRNA dysregulation in the prognosis of TSCC.

A search strategy combining the terms (Tongue Squamous Cell Cancer OR TSCC OR neoplasm OR cancer OR malignancy OR malignant neoplasm OR malignant neoplasms OR neoplasia) AND (prognosis OR prognostic factor OR prognostic factors) AND (MicroRNAs OR Micro RNA OR MicroRNA OR Primary MicroRNA OR Primary miRNA OR RNA, Small Temporal OR Small Temporal RNA OR miRNA OR miRNAs OR pre-miRNA OR pri-miRNA OR stRNA) was developed. Moreover, identified articles were scrutinized separately by reviewing the content of abstracts and full texts for eligible articles meeting the inclusion criteria for this meta-analysis.

### Data collection process and data characteristics

Investigators extracted data from studies that met the inclusion criteria and subsequently contrasted, collated and adopted the data mentioned above. If distinctions in data existed, one researcher expressed opinions that played a critical part in data selection. Data extracted from studies that met the inclusion criteria included Kaplan–Meier curves, first author and date, country, study design, number of patients, cut-off between low and high expression, miRNA types, miRNA expression levels and HRs of deregulated miRNA expression (OS, DSS, DFS, RFS, PFS). According to the Tierney method, prognostic index HR data, comprising overall survival (OS), disease-specific survival (DSS), disease-free survival (DFS), recurrence-free survival (RFS) and progression-free survival (PFS), were collected and extrapolated from Kaplan–Meier curves [28].

### Risk of bias in individual studies, summary measures, summary of results, risk of bias between studies, and additional measures

Investigators evaluated the risk of bias in the studies respectively while ensuring the accuracy of the assessments. Under the guidance of reference factors derived from the Newcastle‒Ottawa Scale (NOS), each study was graded from 0 to 9. Those scoring above or equal to 7 were considered high-quality articles, while those scoring below 7 were eliminated from this meta-analysis. Forest plots were used to graphically illustrate the results mentioned above, and inconsistency indexes involving the Higgins index I² were used for evaluation. Software STATA 15.1 and Reviewer Manager 5.4 were used for statistical analysis. Collected data were aggregated to obtain a pooled sensitivity and specificity of OS, DSS, DFS, RFS and PFS. The risk of bias between the studies was assessed graphically through analysis of overlaps of the confidence intervals through the I² inconsistency index (an I² value greater than 50% was statistically significant, and a fixed-effect model was applied in specific cases). By using a funnel plot and Egger’s rank correlation test, publication bias was comprehensively estimated [29]. If the p value of Egger’s test was *N* > 0.05 and the funnel plot was symmetrical, there was no obvious publication bias in this meta-analysis. Subsequently, we carried out sensitivity analysis to accurately judge the heterogeneity of the selected studies.

## Results

### Study characteristics

**Fig. 1:**
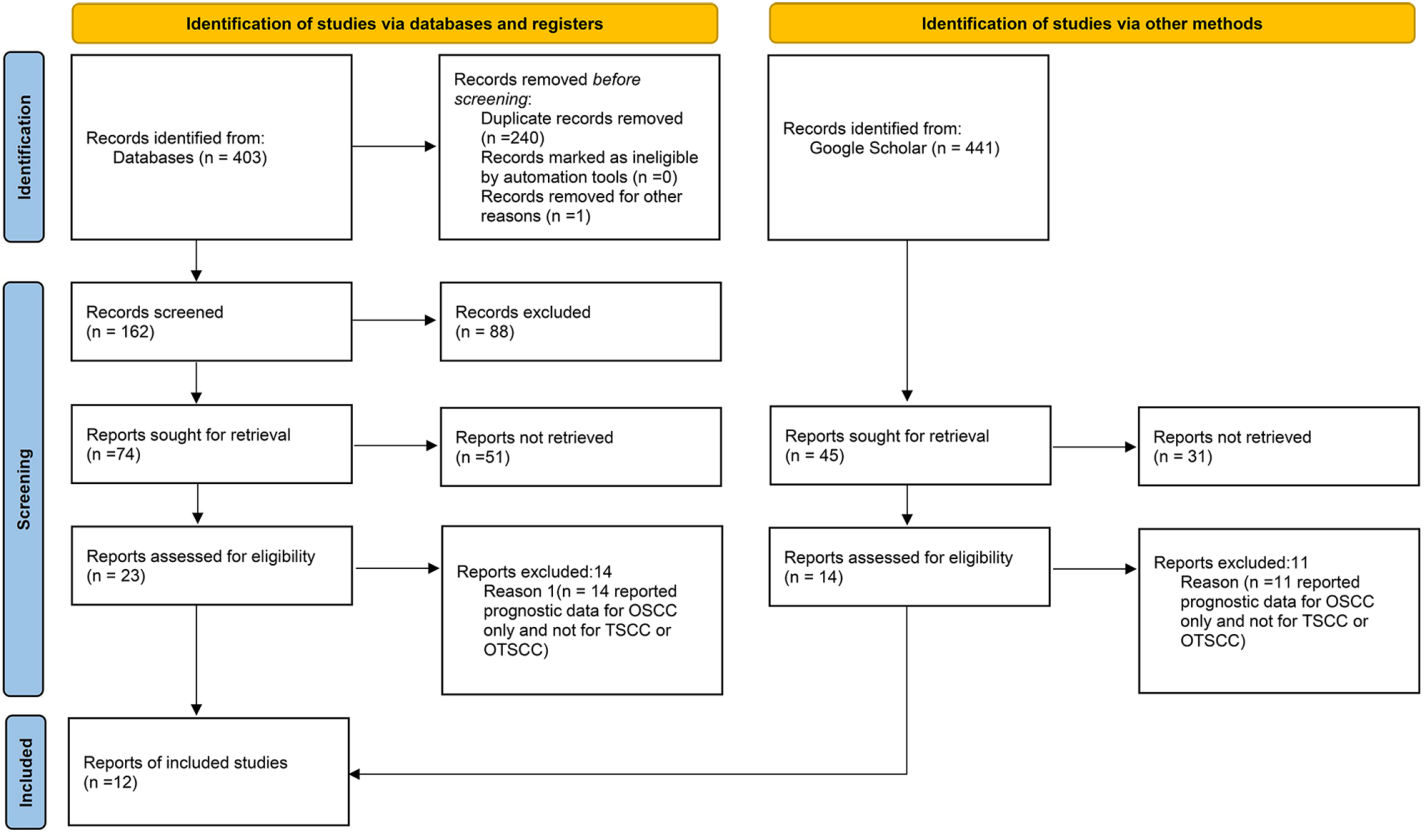
Flow diagram of studies selection process according to PRISMA guidelines

A total of 403 bibliographic citations were initially identified, 240 were excluded due to repetition, and 140 others were excluded according to the eligibility criteria. Fourteen studies reported prognostic data for OSCC only and thus were excluded. Moreover, another 3 articles from Google Scholar that met the inclusion criteria were additionally included in the meta-analysis. In the end, 12 articles were included in our study and 11 of these articles were included in this meta-analysis (Fig. 1).

### Data characteristics

According to the characteristics of the extracted data mentioned in the Data Collection Process and Data Characteristics section, all the extracted data are shown in Table 1. All 12 included retrospective clinical studies with a follow-up period of 3 to 15 years and a total of 970 TSCC patients were published from 2009 to 2020. The studies by Jia et al. (2013) [30], Supic et al. (2018) [31], Zheng et al. (2016) [32], Jia et al. (2014) [33], Li et al. (2009) [34], Maruyama et al. (2018) [35], Xie et al. (2016) [36], Berania et al. (2017) [37], Jia et al. (2015) [38], W. Chen et al. (2019) [39], and S. Chen et al. (2019) [40] reported OS as a prognostic parameter; Kawakita et al. (2014) [41]used DSS; PFS was selected as another prognostic indicator by Berania et al. (2017) [37], and Maruyama et al. (2018) [35] regarded DFS as a prognostic index, while RFS was indicated by Supic et al. (2018) [31].

**Table 1:** Main characteristics extracted from the selected studies

First author, Date	Country	Study design	biological source	Number of patients	Cut Off	MiRNA type	MiRNA expression	HR of deregulated miRNA expression (OS, DSS, DFS, RFS, PFS)
Jia(2013)	China	RT	Tissue	81	Median	miR-195	Downregulation	OS: HR,1.98 (1.28–3.31)
Supic(2018)	Serbia	RT	Tissue	60	ROC analysis, Cutoff = 2.407	miR-183, miR-21	Upregulation	miR-183-OS: HR,5.666(1.708–18.791) miR-21-OS: HR,2.002 (0.904–4.434) miR-183-RFS: HR,1.868(0.924–3.776)
Zheng(2016)	China	RT	Tissue	72	Score ≥ 2	miR-21	Upregulation	OS: HR,1.32 (1.09–2.56)
Jia(2014)	China	RT	Tissue	76	Tertile	miR-26a	Downregulation	OS: HR,2.74 (1.44–5.21)
Li (2009)	China	RT	Tissue	103	Median	miR-21	Upregulation	OS: HR, 2.06 (1.21–3.51)
Kawakita(2014)	Japan	RT	Tissue	79	Score ≥ 1	miR-21	Upregulation	DSS: HR, 1.19 (0.71–1.9)
Maruyama(2018)	Japan	RT	Tissue	50	Median	miR-196a-5p	Upregulation	OS: HR, 1.01(1.21–5.19); DFS: HR, 3.06 (1.32–7.12)
Xie(2016)	China	RT	Tissue	100	Score ≥ 1	miR-320a	Downregulation	OS: HR,1.98(0.939–3.04)
Berania(2017)	Canada	RT	Tissue	58	median	miR-18a, miR-548b	miR-18a-Upregulation miR-548b-Downregulation	miR-18a-OS: HR,4.20(1.55–13.23) miR-18a-PFS: HR,6.89 (1.97–29.86) miR-548b-OS: HR,3.55(1.42–10.82)
Jia(2015)	China	RT	Tissue	105	median	miR-375	Downregulation	OS: HR,2.01(1.20–3.36)
W. Chen(2019)	China	RT	Tissue	126	median	miR-5787	Downregulation	OS: HR,1.976(1.359–2.873)
S. Chen(2019)	China	RT	Tissue	60	median	miR-611	Upregulation	OS: HR,2.19(0.92–5.18)

The abbreviation S. W. indicates that the authors share the last same but different first name

### Risk of bias within studies

The risk of bias was assessed through factors derived from the NOS. According to the NOS guidelines, each study was graded from 0 to 9, and those scoring above or equal to 7 were considered high-quality studies (Table 2).

**Table 2:** The Newcastle-Ottawa Scale (NOS) used for evaluating the risk of bias within Studies

First Author, Date	Quality evaluation	Representativeness of exposed cohort	Selection of non- exposed cohort	Ascertainment of exposure	Outcome not present before study	Comparability	Assessment of outcome	follow-up long enough	Adequacy of Follow Up
Jia (2013)	8	1	1	1	0	2	1	1	1
Supic (2018)	8	1	1	1	0	2	1	1	1
Zheng(2016)	7	1	1	1	0	1	1	1	1
Jia (2014)	8	1	1	1	0	2	1	1	1
Li (2009)	8	1	1	1	0	2	1	1	1
Kawakita(2014)	8	1	1	1	0	2	1	1	1
Maruyama (2018)	7	1	0	1	0	2	1	1	1
Xie (2016)	8	1	1	1	0	2	1	1	1
Berania(2017)	8	1	1	1	0	2	1	1	1
Jia (2015)	7	1	1	1	0	2	1	0	1
W. Chen (2019)	8	1	1	1	0	2	1	1	1
S. Chen (2019)	7	1	1	1	0	2	1	0	1

Additionally, GRADE pro-GDT was used to assess the quality of OS prognostic outcome (Table 3). The results indicated that the quality of the evidence is critical for the outcome.

**Table 3:** Evaluation of GRADE pro-GDT

Certainty assessment							№ of patients		Effect	Certainty	Importance
№ of studies	Study design	Risk of bias	Inconsistency	Indirectness	Imprecision	Other considerations	Intervention	Control	Relative (95% CI)		
13	Observational studies	not serious	not serious	not serious	not serious	all plausible residual confounding would suggest spurious effect, while no effect was observed dose response gradient	495/891 (55.6%)	396/891 (44.4%)	**HR 1.34** (1.25 to 1.44)	⨁⨁⨁⨁^1^ High	CRITICAL

^1^Certainty: ⨁⨁⨁⨁ High

The HR of OS-1.34 (1.25 to 1.44) indicated higher levels of different miRNAs expressions in TSCC patients, demonstrating great statistical significance in prognosis of TSCC patients

### Correlation of miRNA expression with survival outcomes

HR extracted from the included studies was the primary index for assessing TSCC patients with up-regulated and down-regulated miRNA expression. A fixed model was chosen to calculate the pooled HR value (95% CI).

**Fig. 2:**
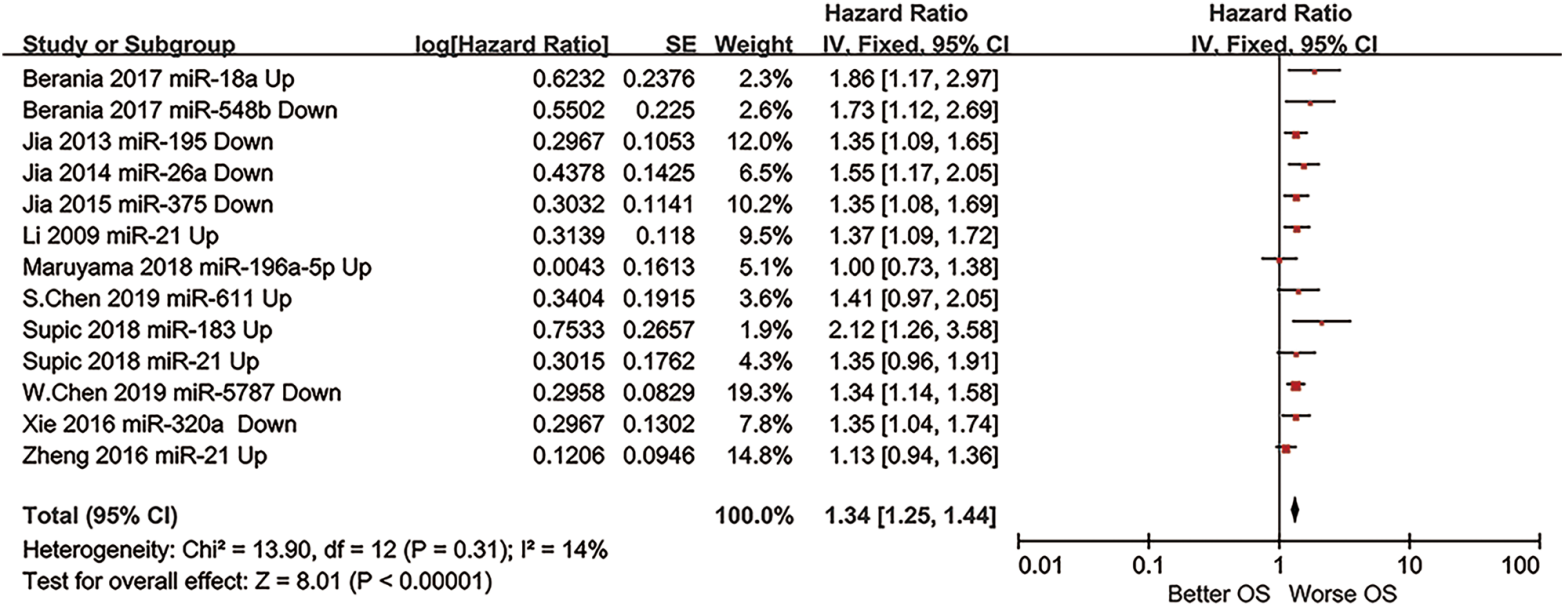
Forest plot of the OS prognostic outcome of the meta-analysis

The prognostic outcome OS from different miRNA expression levels was represented by a forest plot (Fig. 2), showing a pooled HR of 1.34 (1.25–1.44); heterogeneity of Chi^2^ = 13.90; df = 12 (*p* = 0.31); I^2^ = 14%; and test for the overall effect of Z = 8.01 (*p* < 0.00001).

Moreover, even if the prognostic outcomes of DFS PFS RFS DSS were excluded from our meta-analysis in reason of only one study was included in each group, these outcomes still indicated great statistical significance in demonstrating miRNA as a plausible prognostic biomarker for TSCC patients.

**Fig. 3:**
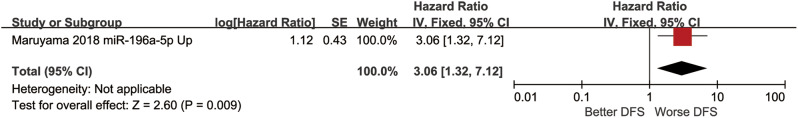
Forest plot of DFS outcome

DFS was regarded as a prognostic indicator, the HR was 3.06 (1.32–7.12) with a test for the overall effect of Z = 0.19 (*p* = 0.009) (Fig. 3).

**Fig. 4:**
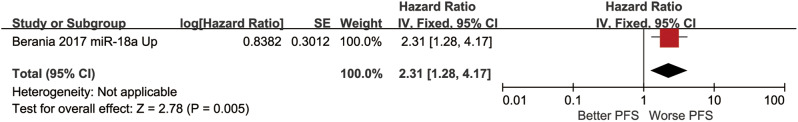
Forest plot of PFS outcome

PFS was considered as a prognostic index, whose data were collected from the study, the HR was 2.31 (1.28,4.17), and the test for the overall effect was Z = 2.78 (*p* = 0.005) (Fig. 4).

**Fig. 5:**
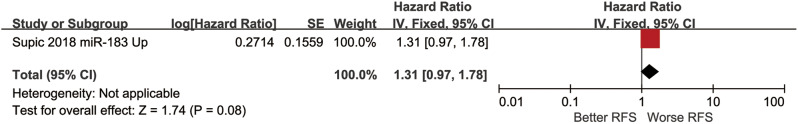
Forest plot of RFS outcome

The HR of another prognostic indicator RFS was 1.31 (0.97,1.78), and the test for the overall effect was Z = 1.74 (*p* = 0.08) (Fig. 5).

**Fig. 6:**
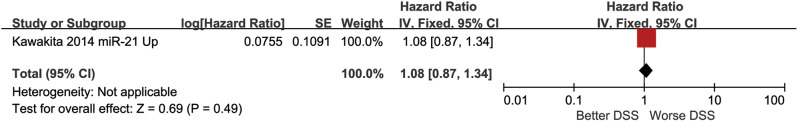
Forest plot of DSS outcome

The HR of DSS prognostic indicator was 1.08 (0.87–1.34) and a test for the overall effect was Z = 0.69 (*p* = 0.49) (Fig. 6).

### Heterogeneity and sensitivity analysis

**Fig. 7:**
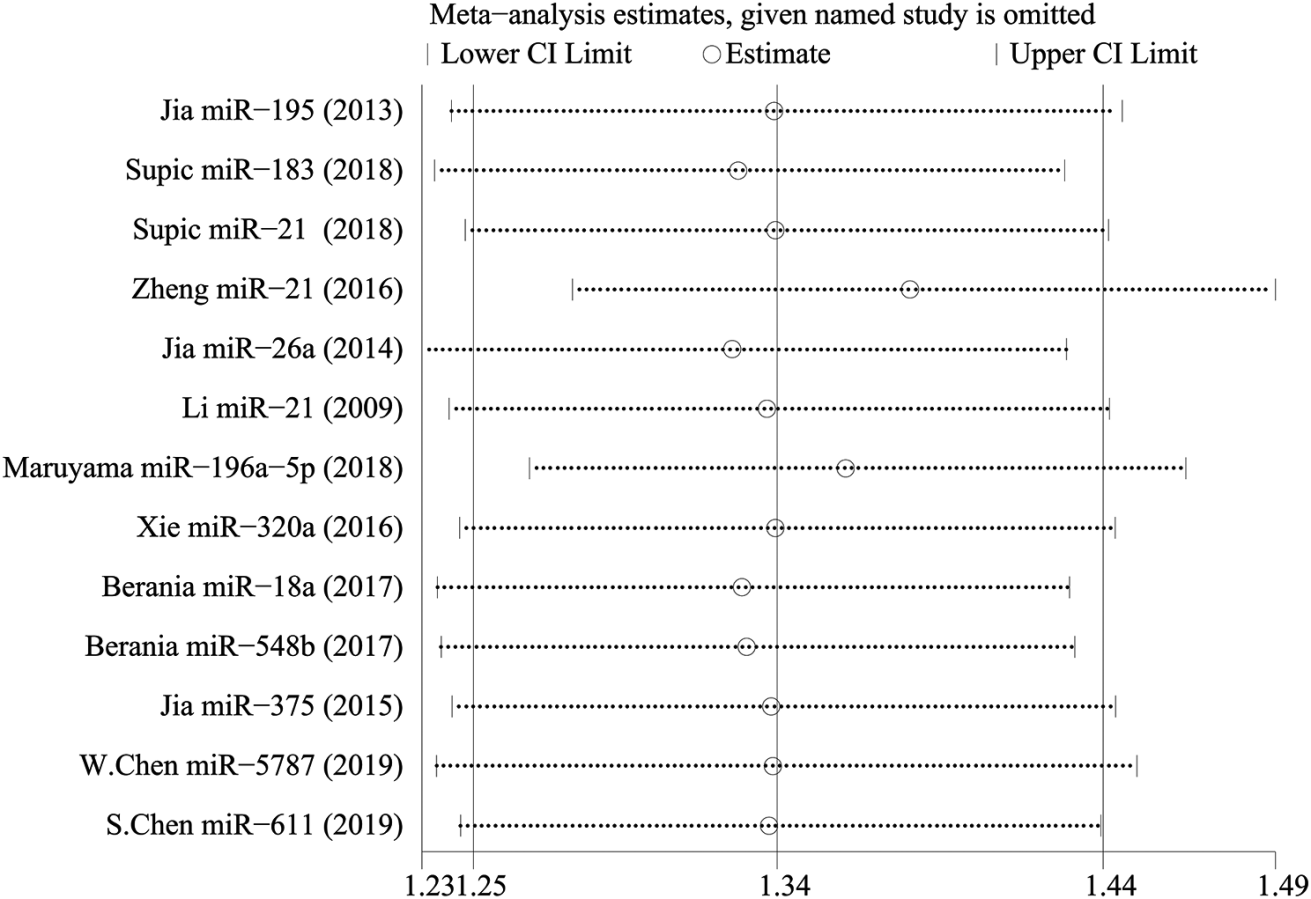
Outcome of sensitivity analysis to assess the heterogeneity in this meta-analysis

There was no significant heterogeneity in this meta-analysis. To ascertain the effect of each included study on the pooled HR, we conducted a sensitivity analysis. With each study excluded successively, the remaining studies were meta-analysed, and the results of pooled HRs were observed. Eventually, the results suggested that no study had an influence on the pooled HR, which means that the results of the meta-analysis were statistically stable (Fig. 7).

### Publication Bias

**Fig. 8:**
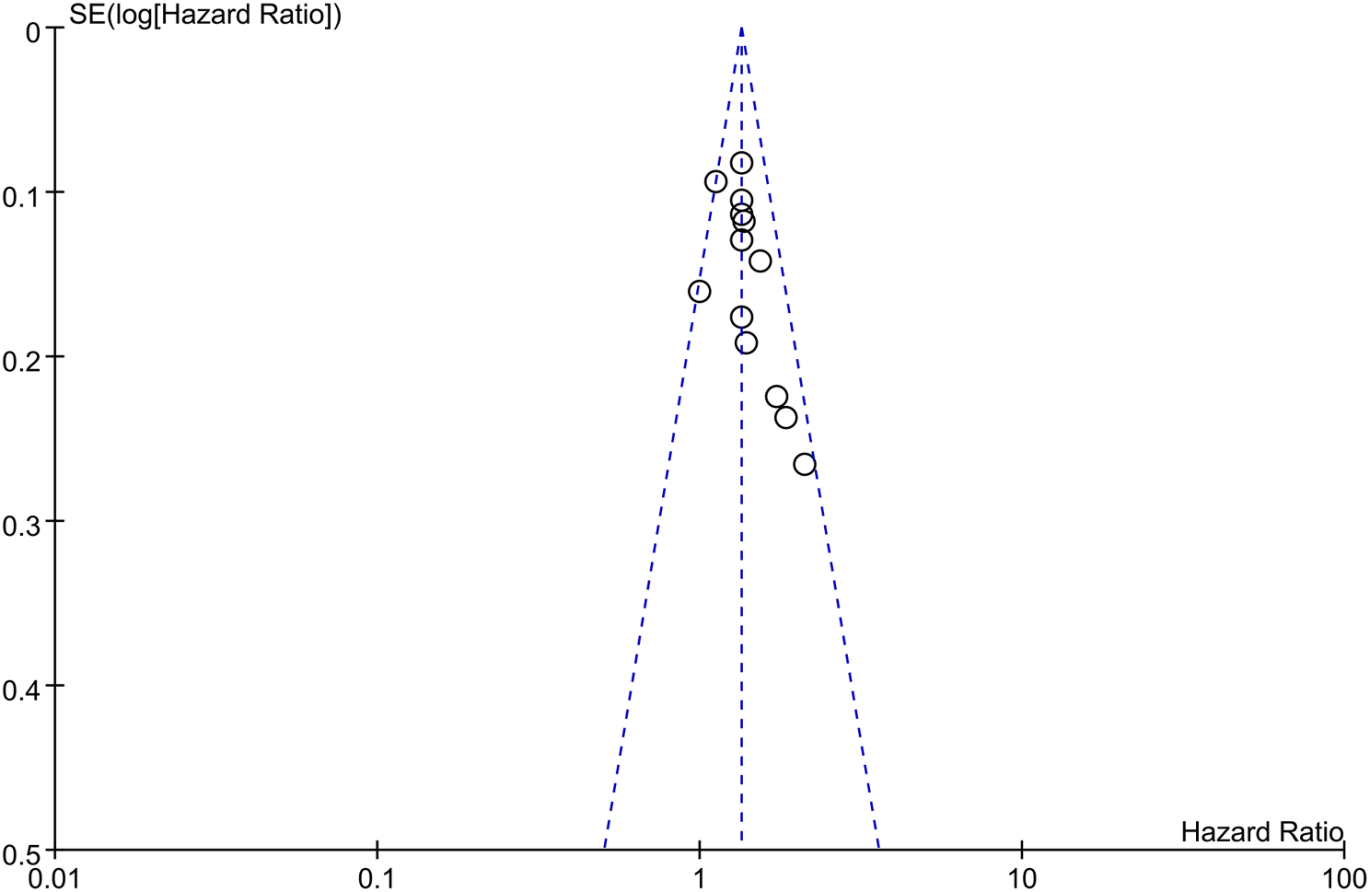
Funnel plot for the main outcome in the fixed-effects model

**Fig. 9:**
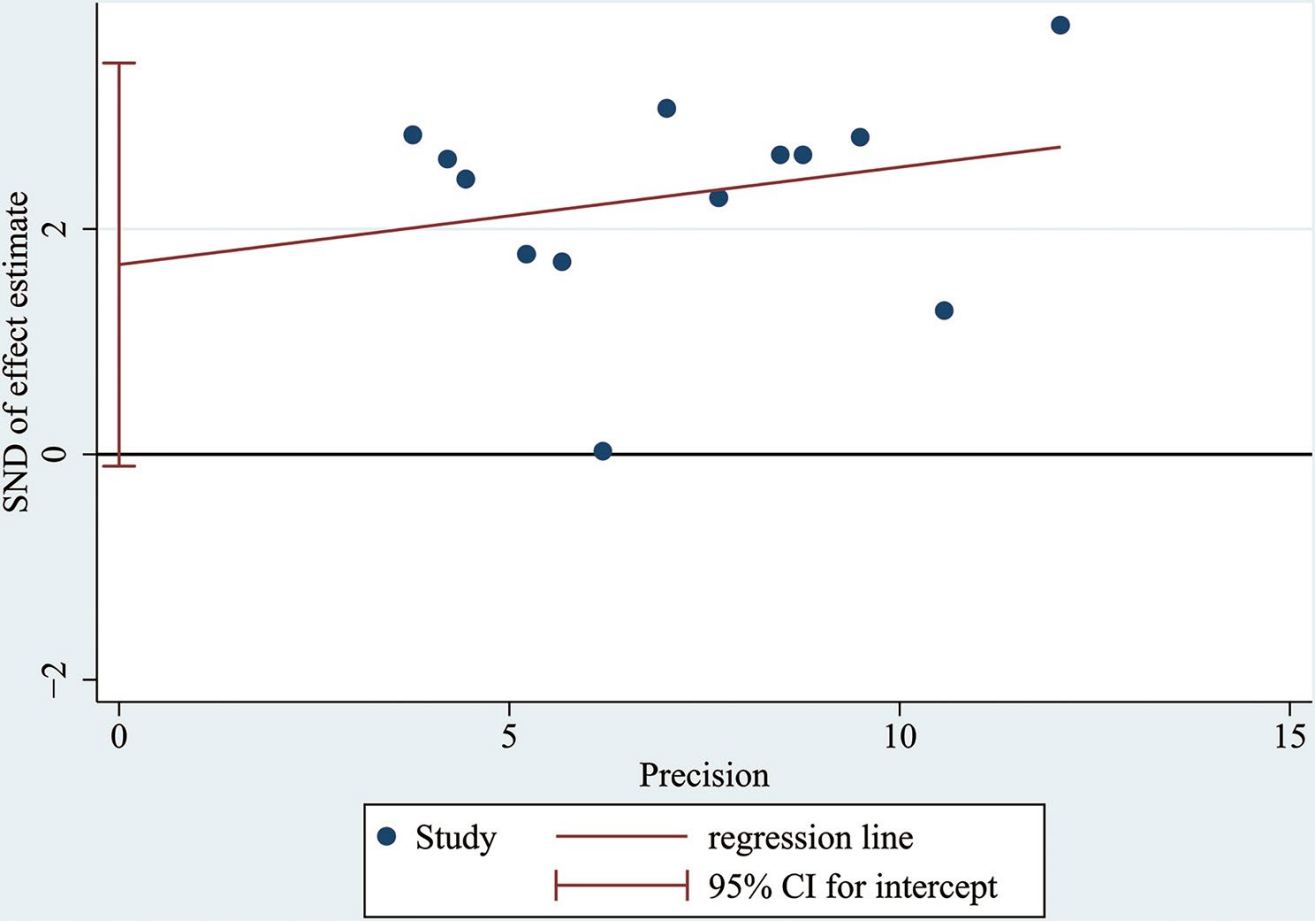
Egger’s test conducted to estimate publication bias; *p* = 0.063 (≥ 0.05)

A funnel plot and Egger’s test were conducted to assess publication bias. According to the figure displayed, there was no visible asymmetry shown by the funnel plot (Fig. 8). To be more accurate, Egger’s test was performed to assess publication bias, and the results showed *p* = 0.063 (≥ 0.05). Thus, there was no statistically significant indication of publication bias, which verified that our results were relatively stable (Fig. 9).

## Discussion

Of the different OSCC subtypes, TSCC has the highest morbidity rate [1], with a low 5-year survival rate despite advancements in treatment during recent years [2, 4]. To extend and improve the quality of patients’ lives, studies have investigated novel indicators targeting tumorigenesis and tumour metastasis, including but not limited to centromeric probes, gene expression profiling and standard karyotyping [5]. Among these possible methods, miRNAs providing regulatory mechanisms based on their expression level have become a hotspot among academic research. Previous studies confirmed down-regulated miRNAs (miR-26a, miR-137, miR-139-5p, miR-143-3p, miR-184, miR-375, miR-32, miR-20a, miR-16 and miR-125b) and up-regulated miRNAs (miR-21, miR-31, miR-134, miR-155, miR-196a, miR-196b, miR-218, miR-455-5p, miR-372 and miR-373) in TSCC prognosis [42–45]. In general, miRNAs are expected to be advanced epigenetic biomarkers in the prognosis and optimized therapy of TSCC [46–48].

In this meta-analysis, the researchers included 11 articles and 891 patients. The investigators further summarized and proposed miRNA expression as a prognostic biomarker for TSCC survival. From the researchers’ review, the pooled HR of the prognostic outcome overall survival (OS) was 1.34 (1.25–1.44), elucidating the potential prognostic value of miRNA expression in TSCC patients. This meta-analysis demonstrated the high feasibility of applying miRNA as a prognostic biomarker in TSCC. Nevertheless, further investigation and improvement are needed.

Based on the included studies and forest plots mentioned above, the expression levels of miRNA-18a, miRNA-611, miRNA-21, miRNA-183, and miRNA-196a-5p significantly increased, whereas those of miRNA-548b, miRNA-5787, miRNA-195, miRNA-26a, miRNA-357, and miRNA-320a apparently decreased, revealing that abnormal expression of miRNAs could be considered a signal for poor prognosis. In addition, miRNA-21, miRNA-183 and miRNA-18a have already been suggested in multiple studies as potential prognostic indicators of head and neck squamous cell carcinoma or oral squamous cell carcinoma [44, 45]. Furthermore, recent studies have made new progress in considering miRNA-5787 and miRNA-357 as TSCC prognostic biomarkers [38, 39]. Altogether, this review offers a new perspective on addressing refractory TSCC prognosis.

With respect to the statistical process, this meta-analysis applied the NOS to assess the quality of selected retrospective cohort studies. According to the criteria of this protocol, 11 included studies scored more than 7 points, which means that they had a low risk of bias and high quality. Pooled HRs (95% CI) of prognostic factors extracted from the forest plot was OS-1.34 (1.25–1.44), which implied low heterogeneity. Additionally, researchers determined that the data collected were credible and accurate enough to demonstrate miRNA as a plausible prognostic biomarker for TSCC patients via sensitivity analysis and Egger’s test.

Nevertheless, the limitations of this meta-analysis should not be ignored. First, it is inevitable that the quantity of patients included is relatively insufficient since supplementary cases could enhance credibility. Second, due to the intrinsic deficiency of the Kaplan‒Meier curve, it is difficult for researchers to verify part of the accuracy of the initial results since extraction based on image capture could not be perfected. Third, due to limited resources in online databases, the cases contained were entirely extracted from only East Asia and American countries, which obviously led to regional limitations. Hence, patients from other nations could not completely benefit from the current results. Therefore, these problems must be scientifically addressed in future studies.

During the information retrieval process, the investigators noted that exosome research, especially involving miRNAs, has drawn much attention from academic communities and has even been experimentally used as diagnostic and prognostic tools in several types of malignant tumors [49, 50]. In comparison with other advanced techniques for prognosing TSCC, miRNA expression levels provide an original method for preventing recurrence and evaluating therapeutic effects. Not only could miRNA expression prospectively be used as a biomarker of residual or recrudescent TSCC malignant tissue, but it could also be utilized to make more accurate prognoses prior to clinical manifestations, which has significance for the timely initiation of adequate treatment. In summary, this meta-analysis aimed to contribute to the development of novel treatments and provide persuasive references for practical clinical applications and therapeutic guidance to some extent.

## Conclusion

In conclusion, in this meta-analysis, the data extracted from the OS prognostic outcome suggest that alterations, including up-regulation and down-regulation of miRNA expression levels, could be used as promising prognostic factors of TSCC. According to the figures and tables presented above, the down-regulated miRNAs correlated with poor prognosis are miR-195, miR-26a, miR-320a, miR-548b, miR-375 and miR-5787, while the up-regulated miRNAs are miR-183, miR-21, miR-196a-5p, miR-18a, and miR-611. Given the limitations of the existing studies, it is necessary to conduct repetitive studies with more statistical tests and large-scale experiments that include patients from various regions and nations as well as various age groups. Consequently, this meta-analysis could provide references and prerequisites for further clinical trials and therapeutic applications.

## Data Availability

All data generated or analysed during this study are included in this published article.

## Abbreviations

miRNA: microRNA
HR: hazard ratio
OSCC: Oral squamous cell carcinoma
NOS: Newcastle‒Ottawa Scale
WHO: World Health Organization
OS: overall survival
DSS: disease-specific survival
DFS: disease-free survival
RFS: recurrence-free survival
PFS: progression-free survival
